# Marine-Based Nutraceuticals: An Innovative Trend in the Food and Supplement Industries

**DOI:** 10.3390/md13106336

**Published:** 2015-10-14

**Authors:** Hafiz Ansar Rasul Suleria, Simone Osborne, Paul Masci, Glenda Gobe

**Affiliations:** 1 School of Medicine, the University of Queensland, Translational Research Institute, Kent Street, Woolloongabba, Brisbane 4102, Australia; E-Mails: hafiz.suleria@uqconnect.edu.au (H.A.R.S.); p.masci@uq.edu.au (P.M.); 2 CSIRO Agriculture Flagship, 306 Carmody Road, St Lucia, QLD 4067, Australia; E-Mail: simone.osborne@csiro.au

**Keywords:** bioactive molecules, marine nutraceuticals, functional foods, supplements and health perspectives

## Abstract

Recent trends in functional foods and supplements have demonstrated that bioactive molecules play a major therapeutic role in human disease. Nutritionists and biomedical and food scientists are working together to discover new bioactive molecules that have increased potency and therapeutic benefits. Marine life constitutes almost 80% of the world biota with thousands of bioactive compounds and secondary metabolites derived from marine invertebrates such as tunicates, sponges, molluscs, bryozoans, sea slugs and many other marine organisms. These bioactive molecules and secondary metabolites possess antibiotic, antiparasitic, antiviral, anti-inflammatory, antifibrotic and anticancer activities. They are also inhibitors or activators of critical enzymes and transcription factors, competitors of transporters and sequestrants that modulate various physiological pathways. The current review summaries the widely available marine-based nutraceuticals and recent research carried out for the purposes of isolation, identification and characterization of marine-derived bioactive compounds with various therapeutic potentials.

## 1. Background

The concept of “nutraceutical” was introduced by Stephen DeFelice in 1989, by combining the terms “nutrition” and “pharmaceutical”. The term refers to raw foods, fortified foods or dietary supplements containing biologically active molecules, also known as bioactive molecules [1] that provide health benefits beyond basic nutrition [2]. These bioactive compounds include certain polysaccharides, peptides, phytochemicals, vitamins, and fatty acids that are naturally present in foods, can be added to foods producing fortified or functional foods or can be formulated into dietary supplements. These bioactive molecules can be obtained either by extraction from natural sources or by chemical and biotechnological synthesis [3].

Marine ecosystems have a high diversity of living organisms compared to terrestrial ecosystems providing numerous resources for human nutrition and health [4]. Marine invertebrates are a diverse group with habitats in all ocean ecosystems, ranging from the intertidal zone to the deep sea environment. Organisms belonging to marine invertebrates are composed of different taxonomic groups, which can be classified into several major phyla, namely, Porifera (sponges), Cnidaria (corals, sea anemones, hydrozoans, jellyfish), Annelida (Polychaetes, marine worms), Bryozoa (moss animals or sea mats), Mollusca (oysters, abalone, clams, mussels, squid, cuttlefish, octopuses), Arthropoda (lobsters, crabs, shrimps, prawns, crayfish), and Echinodermata (sea stars, sea cucumbers, sea urchins) [5]. This diverse group also includes macroalgae, microalgae, bacteria, cyanobacteria, certain fish species and crustaceans that produce secondary metabolites as an adaptation to their hostile marine environment.

Marine sources have received great attention recently; research on marine-derived molecules has discovered new bioactive compounds with important properties increasing their applicability as nutraceuticals in the food and supplement industries. Most notably Hippocrates, the “father of modern medicine”, is recorded as describing the therapeutic effects of various marine invertebrates and their constituents on human health [6]. Marine invertebrates account for nearly 40% of global fisheries and seafood obtained from these sources are highly recognized for health benefits attributed to higher amounts of polyunsaturated fatty acids, various peptides, minerals (selenium and iodine) and other bioactive compounds such as carotenoids and taurine [7]. Numerous molecules with biomedical functions are also supplied by marine ecosystems and are used as pharmaceuticals.

## 2. Nutraceuticals in the Global Market

The global nutraceutical market comprised of functional foods and beverages and dietary supplements, was valued at around $250 billion in 2014. Consumer demand for nutraceuticals is rapidly increasing with the market expected to reach around $385 billion by 2020 [8].

Nutraceutical products have a highly competitive market and price compared with conventional therapies; in particular their market share is increasing in many regions including the United States of America (USA), Europe, Japan, Asia Pacific, Middle East and Latin America. A recent analysis of the global industry [9] offers a detailed report of the industry structure, market drivers, trends, issues and competition. The global market is dominated by the USA, Europe and Japan who contribute more than 85% of the market. It is predicted that these three regional markets will remain at the forefront of the nutraceutical industry both as producers and consumers due to greater product awareness, higher income levels and a preference for nutraceutical products, preventive medicine and self-treatment. It is estimated that the Asia Pacific region, particularly China and India, will experience significant growth in the long term, because of increasing awareness, income and wealth.

In the regions where nutraceutical product consumption is high, a number of dietary supplements, medical foods, food complements, and functional foods have invaded the food and health market with the aim of maintaining health and preventing disease. Within these regions, consumption rates of these products vary with the country, age and sex of the population. For example, consumption of nutraceutical products is higher in the USA [10] than any other region in the world where approximately 40% of adults consume nutraceutical products. In a Spanish population of 6352 males and females aged 35 to 80 years, 9% were consumers of dietary supplements (mainly vitamins and minerals). Amongst these consumers, 72% were women aged 35 to 49 years with a higher educational level and greater adherence to the Mediterranean diet pattern than non-consumers [11].

It has also been found that intake of nutraceutical products varies with lifestyle, for example increased physical activity and high fruit and dietary fiber intake is associated with the use of nutraceutical products, whereas nutraceutical product consumption decreases with obesity, smoking and increased intake of dietary fat [12]. Thus individuals with healthy lifestyles are more commonly the users of nutraceutical products. The intake of these types of products is also of special relevance for aging populations [13]. Elderly people are more susceptible to micronutrient deficiencies due to a variety of factors including social, physical, economic and emotional obstacles to eating. In response to the large and increasing populations of elderly individuals, there is an important need to encourage and maintain a healthy lifespan and prevent chronic illnesses associated with aging [14]. In the United Kingdom (UK) the Mintel survey on Vitamins and Minerals supplements observed that 25% of all adults were convinced of the benefit of the nutraceutical products and that usage varied with age; 86% of 55 year olds were found to use nutraceutical products compared with 65% of 20–24 year olds [15].

Marine nutraceutical products represent a large portion of the global market and are derived from a diverse range of sources that provide a myriad of bioactive molecules. These sources, as well as the constituent bioactive molecules, are discussed in the following sections and summarized in the Table 1.

**Table 1:** Marine bioactive molecules: sources, applications and health perspectives.

Category	Bioactive Molecules	Applications	Major Marine Sources	Health Perspectives	References
Protein and Peptides	Collagen	Edible coating in meat industry (e.g., sausages)	Fish (albacore tuna, silver-line grunt, brown-backed toadfish, hake, trout, lingcod, catfish, rainbow trout, yellow sea bream and common horse mackerel *etc.*).	Anti-oxidant, anti-hypertensive and anti-skin-aging activities.	[16,17]
	Gelatin	Stabilizer, texturizer, or thickener in ice cream, jam, yogurt, cream cheese, margarine, confectionaries, utilized in low fat foods and clarifiers	Fish, especially cold-water (Pollock, cod, haddock, hake and cusk)	It has been shown to prevent and treat chronic atrophic gastritis	[18]
	Albumin	Whipping, suspending, or stabilizing agent	Mollusks, crustaceans, low-fat fish	Anticoagulant and antioxidant properties	[19]
Poly-Saccharides	Carrageenan	Gel formation and coatings in the meat and dairy industry	Macroalgae e.g., *K. alvarezii*, *E. denticulatum* and *B. gelatinum*	Anti-HIV activity and anticoagulant properties	[20]
	Agar agar	Gel formation and food gums	Red Alga is a main source of agar agar like *Gelidium*, *Gracilaria*, *Hypnea* and *Gigartina*		[21]
	Fucans and fucanoids	Nutraceutical supplements	Cell walls of brown algae, sea urchin eggs, sea cucumbers	Anticoagulant, antiviral, antithrombotic, proliferative and anti-inflammatory	[22]
	Chitin, chitosan, and derivatives	Gelling agents, edible protective films, clarification and de-acidification of fruits	Shrimp, crab, lobster, prawn and krill	Increase dietary fiber, reduce lipid absorption, antitumor, bactericidal and fungicidal activities	[23]
Fatty acids	Omega-3 fatty acids	Nutraceuticals (fish oil and capsules), fortification of livestock, feed and infant formula	Almost all marine sources	Numerous health benefits (e.g., visual and neurodevelopment, reduce risk of cardiovascular problems, ameliorate diseases such as arthritis and hypertension)	[24]
Phenolic compounds and other pigments	Phlorotannins	Active ingredients in the nutraceuticals	They are the most abundant polyphenols found in the marine brown algae	Antioxidant activity	[25]
	Carotenoids: β-carotene, and lutein	Natural food colorings, nutraceutical agents, farmed salmon pigmentation	*Dunaliella salina*, *Sarcina maxima*, *Chlorella protothecoides*, *Chlorella vulgaris* and *Haematococcus pluvialis*	Vitamin A precursors, antioxidants, anti-carcinogenic and anti-inflammatory	[26]
	Chlorophylls	Natural food and beverage colorants	*S. platensis* and *A. flos-aquae*	Anticancer activity, natural source of pigmentation	[27]
Marine enzymes	Gastric proteases; pepsins, gastricsins and chymosins	Cold renneting milk and fish feed digestion aid	Various fish body viscera like atlantic cod, carp, harp seals, and tuna *etc.*		[28]
	Serine and cysteine proteases	Preventing unwanted color changes in food products, meat tenderizing, curing of Herring, squid fermentation	Crustaceans, mollusks and short-finned squid		[28]
	Lipases	Numerous uses in the fats and oils industry	Atlantic cod, seal, salmon, sardine, Indian mackerel and red sea bream		[28]
	Transglutaminase	Creates protein cross-links to improve rheological properties of gels, *i.e.*, surimi, gelatin	Red sea bream, rainbow trout, atka mackerel, walleye, Pollock liver and scallop		[29]
Vitamins and Minerals	Fat and water soluble vitamins, iron, iodine, manganese and zinc	Food, Pharma and nutraceutical industries	Almost all marine sources. Seaweeds are rich sources of vitamins and minerals	Vitamins and minerals perform many essential functions in the body, for example, they provide transport inside cells and also serve as cofactors during metabolic processes	[30]

## 3. Marine Sources of Bioactive Molecules

Marine organisms represent a valuable source of bioactive compounds. The biodiversity of the marine environment and the associated chemical diversity constitute practically an unlimited resource for the development of new bioactive products [31]. Marine organisms such as sponges, tunicates, bryozoans, molluscs, bacteria, microalgae, macroalgae and cyanobacteria have recently been utilized for biotechnology. Compounds produced from these organisms are effective as therapeutics for infectious and non-infectious disease, with a high specificity for target molecules, usually an enzyme.

### 3.1. Marine Algae

In the nutraceutical industry marine algae are used as sources of food and food ingredients [32]. Microalgae and macroalgae are the two main categories of algae and both have applications as nutraceuticals.

Microalgae, the most primary and simply-organized members of marine plant life, are rich sources of food ingredients, such as β-carotene, Vitamins C, A, E, H, B1, B2, B6 and B12, astaxanthin, polysaccharides and polyunsaturated fatty acids [33,34,35]. As such, bioactive molecules from microalgae are commercially produced, used as food additives and also incorporated into infant milk formulations and dietary supplements [36].

Macroalgae, also called seaweed, are the most popular type of algae in the nutraceutical industry as it provides a great variety of food and food ingredients especially in Asian countries like Korea, Japan and China [37]. It is estimated that the annual production value of macroalgae is US$5 billion in 2007 [38,39]. Agarose is one of the main products from macroalgae, however macroalgae are also sources of metabolites and natural products with unique nutritional and therapeutic properties. These metabolites and natural products include proteins, furanone, polyunsaturated fatty acids, l-α kainic acid, phenotics, pigments, phlorotannins, phycocolloids (carrageenan and agar) and minerals [40].

Macroalgae are an important source of essential nutrients and new bioactive molecules for human nutrition [41]. For example, red and brown seaweeds are alternative sources of vitamins, minerals, and proteins [42] and are good sources of essential fatty acids [43]. They have been used to prepare bioactive peptides and to improve protein digestibility [44]. Recently, antihypertensive bioactive peptides have been isolated that may act as angiotensin-converting enzyme (ACE) inhibitors [45]. Macroalgae are also rich sources of insoluble and soluble dietary fiber [46] as they are not digested by enzymes in the gut [47] and are mainly composed of indigestible sulfated polysaccharides [43]. Examples of structural and storage polysaccharides found in red and brown seaweeds include fucan, agar, laminaran, carrageenan and alginate [48]. The alginates from brown seaweeds are utilized as hydrocolloids due to their biological activity [49]. Food and cosmetics industries use fucans from brown seaweeds. However, it is likely that all bioactive molecules from algae have not yet been identified and that molecules from marine algae may provide different health benefits and biological activities [50].

### 3.2. Marine Fish

Fish occupy the highest position in marine animal consumption and are important to the world economy. In 2012 fish provided approximately 16% of the world’s protein requirements with herring, salmon, cod, flounder, tuna, mullet and anchovy being the most common species of fish used for food [51]. One of the largest commercially canned fishery products in the world is tuna (for example, *Thunnus Obesus*). According to the Food and Agriculture Organization (FAO) of the United Nations (2010), the total catch of the commercial tuna species increased from 162,980 metric tons in 1950 to more than 4.2 million metric tons in 2007 [52]. The nutritional benefits of fish consumption are due to the presence of proteins, unsaturated essential fatty acids, minerals (for example, calcium, iron, selenium and zinc), and vitamins, namely Vitamin A, B3, B6, B12, E and D. Research has also shown that peptides derived from fermented fish following enzymatic treatment may be useful therapeutics for the treatment of many common acute and chronic diseases such as viral infections, hypertension, cancer and Alzheimer’s disease [53,54]. Fish collagen may also be used in bone treatment as an alternative to mammalian collagen which is known to be immunogenica [55].

### 3.3. Marine Invertebrates

Marine invertebrates, like sponges, molluscs, echinoderms and crustaceans, are sources of bioactive peptides, steroids, terpenoids, strigolactones, alkaloids, ether and phenols [56]. Extracts or compounds isolated from marine invertebrates are known to include antibacterial, antiviral, anthelmintic, antifungal, antihypertensive, anticancer and have immune modulatory properties. A great deal of research has been conducted on these molecules particularly for the development of many new therapeutic agents [57].

### 3.4. Sponges

Sponges are sessile metazoans that consist of a gelatinous material, mesophyl, and are the simplest form of multicellular animals. Their hollow pitcher-like body is reinforced by spicules (a needle-like silica or calcium structure) and spongia (collagen fibers) [58]. There are many chemically-diverse compounds like alkaloids, terpenoids, polyketides, macrolides, polyphenolic compounds, peptides and sterols that are isolated from sponges with the potential to cure various ailments [59].

### 3.5. Molluscs, Echinoderms and Crustaceans

Molluscs, together with echinoderms, have been widely consumed as marine foods and are considered natural functional foods. Extensive research has been performed on bioactive molecules from these sources revealing many significant findings. Bioactive peptides obtained from the fermented blue mussel and oyster sauces significantly decrease hypertension [54] whilst ground abalone and its shells are used for treating eye diseases [51]. Many Asian populations consume cuttlefish, squid, octopus and nautiloids due to their therapeutic effects, for example rickets are cured with the bones of cuttlefish, as well as gastrointestinal disorders and ear inflammation [51].

Crustaceans are the biggest and most economically important class of marine arthropods in the global fisheries markets; they also have significant roles in nutraceutical industries [60]. In particular, crabs, prawns and shrimps have gained great attention due to their effective utilization and health benefits [61]. Nutrient composition of marine crustaceans like shrimp and krill was analysed and found to decrease the total blood lipids in humans, and improve Vitamin A levels, specific proteins, and eicosapentaenoic acid, an omega-3 fatty acids. These are suggested to be used in the development of value-added health food products and for human consumption due to higher nutritional value [62]. 

## 4. Marine Derived Bioactive Components

Marine ecosystems provide a diverse range of bioactive molecules with extensive applications as nutraceuticals in the food and supplement industries. These bioactive molecules can be proteins, peptides, polysaccharides, fatty acids, polyphenols, probiotics, enzymes, vitamins and minerals. The remaining sections of this review discuss the physical and chemical properties of these different molecules and how they contribute bioactivity in the context of nutraceutical applications.

### 4.1. Proteins

Proteins from marine sources including fish (cod, tuna, herring, rout, hake, pollock and haddock), crustaceans, molluscs, extremophiles like Dunaliella, and seaweeds have unique properties such as gel-formation, film and foaming capacity, antioxidant, anticoagulant and antimicrobial activity. For example, collagen, gelatin and albumin are common marine proteins used in foods and are enzymatically hydrolyzed for the production of bioactive peptides which may have the potential to be used as nutraceuticals [54,63]. The marine protein protamine is also used as a natural antibacterial preservative in the food industry.

### 4.2. Peptides

Bioactive peptides are protein fragments ranging in size from 2–20 amino acid residues that may be generated from parent proteins during digestion or processing [64]. Two factors can influence the type of bioactive peptides produced: (1) the primary sequence of the protein substrate; and (2) the specificity of the enzyme(s) used to generate such peptides. Furthermore, bioactive peptides can be generated from proteins using hydrolysis (acid or alkaline), cooking or fermentation. These peptides have a range of bioactivities including antimicrobial, immunomodulatory, antithrombotic, and antihypertensive activity [65]. They are considered highly significant compounds.

Research is being performed to understand peptide structure, composition and sequences. Bioactive peptides have various regulatory functions on specific cellular target formulations [66]. Many researchers have focused on the development of pharmaceutical compounds from marine-derived peptides particularly for ACE inhibition and antihypertensive function [67,68]. Marine proteins from fish, molluscs and crustaceans are amongst the richest sources of bioactive molecules [69]. Fouchereau-Peron *et al.* [70] reported that fish protein hydrolysates have some novel peptides that can bind to cell surface receptors and enhance calcium absorption. The therapeutic application of these peptides is the treatment of osteoporosis and Paget’s disease. Collagen is a valuable part of bovine and porcine meat and is used in different industries like cosmetics, pharmaceutics, food and biomedicine. Meat collagen is an excellent source of bioactive peptides that function as antihypertensives and antithrombotics as well as inhibitors of brush border enzymes like dipeptidyl peptidase-IV [71].

### 4.3. Polysaccharides

There are numerous commercial applications of marine polysaccharides in food, beverages and supplements. Marine polysaccharides, extracted from algae, crustaceans and other marine organisms include fucans/fucanoids, carrageenans, hydrocolloids and glycosaminoglycans. These molecules have many biological functions including antiviral, anticoagulant, antiproliferative, antithrombotic and anti-inflammatory activity [22]. Carrageenans and alginates are linear biopolymers that have been identified as the most abundant polysaccharides found in red and brown algae, respectively [72]. Apart from alginate, brown algae also contain highly complex, sulfated matrix polysaccharides called fucoidans. The complex structure of the fucoidans extracted from different marine species varies in saccharide composition, sulfate content, and positions of sulfate groups, molecular weight, linkage mode, and sequence of saccharide residues [73]. Structural sulfate groups improve the biological properties of fucoidans which enables their application as nutraceuticals in the dairy industry [74]. These marine-derived secondary metabolites also have many human health benefits which enable them to be applied as nutraceuticals.

### 4.4. Fatty Acid

Marine fish species and algae have been identified as sources of polyunsaturated fatty acids which are rich in ω-3 or ω-6 fatty acids. The presence of these unsaturated fatty acids in marine-derived foods increases their applicability as nutraceuticals in the food industry [75]. Marine-based nutraceuticals have many unique features not found in nutraceuticals obtained from terrestrial resources, and this is one of the reasons why they are gaining more attention. The most common sources of marine oils are fungi (Phycomycetes), fish (salmon, tuna, sardines, and herring), microalgae, extremophiles, macroalgae (Bryophyta, Rhodophyta) and krill. Consumption of marine oils provides numerous health benefits like visual and neurodevelopment, amelioration of diseases such as hypertension and arthritis and a reduced risk of cardiovascular problems [24].

### 4.5. Phenolic Compounds and Prebiotics

Phenolic compounds found in marine algae are known mainly as a mechanism of adaptation for oxidative stress [76]. Usually phlorotannins are the most abundant polyphenols found in the marine brown algae whereas flavonoids contribute most to the total phenolic content in green algae. The brown algal phlorotannin profile mainly consists of phloroglucinol, eckol, and dieckol [76]. Antioxidant activity has also been reported from phlorotannins enabling these phenolic compounds to be used as active ingredients in nutraceuticals [25]. Similar to polyphenols, carotenoids, synthesized in certain marine bacteria and algal species, also have antioxidant properties which increase their applicability as nutraceuticals. Carotenoids are lipid-soluble, natural pigments with 40-carbon structures [77]. Different carotenoids are synthesized within the marine organisms, for example, β-carotene, astaxanthin and fucoxanthin are known to have a high antioxidant capacity. Antioxidants have protective roles against excess reactive oxygen species, and also act against oxidative rancidity and peroxidation products like superoxide anions, hydroxyl radicals and hydrogen peroxide (H_2_O_2_) that cause deterioration of some foods. Currently, commercial preparation of β-carotene and astaxanthin is available using *Dunaliella* species and *Haematococcus* species, respectively [74].

Prebiotics are non-digestible, selectively-fermented compounds that stimulate the growth and activity of beneficial gut microbiota which, in turn, confer a health benefit to the host. Usually, prebiotics are oligosaccharides such as chitosan oligosaccharides, while certain other algal polysaccharides are also known to have a prebiotic activity [78]. Bifidogenic benefits have been also reported from the exopolysaccharides produced by marine lactic acid bacteria [79]. Further, the cyanobacterial biomass of *Spirulina platensis* is able to stimulate both *Lactobacillus* and *Bifidobacterium* species, promoting their prebiotic effect. Photosynthetic pigments are also obtained from red and blue-green algae, aquatic plants, microalgae and seaweed. These pigments provide nutraceutical agents, natural food coloring, anti-inflammatory, anticarcinogenic and antioxidant compounds [40].

### 4.6. Enzymes, Vitamins and Minerals

Enzymes have the ability to change other molecules into valuable biotechnological tools that can be used in food and nutraceutical industries. As food ingredients, enzymes can influence factors such as spoilage, storage, processing and safety. Enzymes derived from marine sources are lipase, chitinolytic enzymes, polyphenol oxidase (Catecholase, tyrosinase, cresolase, polyphenolase, catechol oxidase, phendase), and transglutamase, and red algal enzymes involved in a starch degradation pathway (for example, α-1, 4-glucanase). They have excellent chemical, physical and/or catalytic properties compared with their terrestrial counterparts. They also have the ability to be inactivated at moderate temperatures and show high catalytic activity when the temperature is low [80]. Additionally these enzymes are used as food ingredients. They are used in food processing due to their salt tolerance, specificity, diverse properties and high activity at mild pH [81]. Biomolecules such as enzymes isolated from extremophiles can be highly useful in the food industry due to their unique activities under abnormal conditions, and it has been widely accepted that extremophiles have strong potential to be valuable resources for use in biotechnology [82].

Vitmains and minerals perform many essential functions in the body, for example, they provide transport inside cells and also serve as cofactors during metabolic processes. Seaweeds are rich sources of vitamins and minerals including iron, iodine, manganese and zinc [30]. Some types of seaweed could be used as natural sources of iodine [83].

## 5. Conclusions

Marine nutraceuticals are attracting a lot of attention in the food and supplement industries. Marine natural products have been used as a promising source of bioactive molecules to prevent and cure various diseases for centuries. Accordingly, marine organisms are also one of the most important sources of foods with the potential to cure various ailments. Marine food provides proteins, peptides, polysaccharides, polyunsaturated fatty acids, vitamins, minerals and many other bioactive compounds such polyphenols. The current review summarizes marine bioactive molecules and their current and potential utilization in food and supplement industries.
